# Neuroradiological emergency consultations during the first year of the COVID-19 pandemic

**DOI:** 10.1186/s42466-021-00147-8

**Published:** 2021-08-30

**Authors:** Johannes A. R. Pfaff, Marcial E. Harlan, Günter Pfaff, Alexander Hubert, Martin Bendszus

**Affiliations:** 1 Department of Neuroradiology, Heidelberg University Hospital, Im Neuenheimer Feld 400, 69120 Heidelberg, Germany; 2 University Institute for Neuroradiology of the Paracelsus Medical University, Uniklinikum Salzburg, Christian-Doppler-Klinik, Ignaz-Harrer-Straße 79, A-5020 Salzburg, Austria; 3 Visiting Lecturer in Epidemiology and Psychiatric Epidemiology, Protestant University of Applied Sciences Ludwigsburg, Paulusweg 6, 71638 Ludwigsburg, Germany

**Keywords:** COVID-19, Stroke, Transient ischemic attack, Consultations, Computed tomography

## Abstract

**Background:**

Measures taking aim at minimizing the risk of coronavirus transmission and fear of infection may affect decisions to seek care for other medical emergency conditions. The purpose of this analysis was to analyze intermediate-term effects of the COVID-19 pandemic on neuroradiological emergency consultations (NECs).

**Methods:**

We conducted an ambispective study on NEC requests to a university hospital from a teleradiological network covering 13 hospitals in Germany. Weekly NEC rates for prepandemic calendar weeks (CW) 01/2019–09/2020 were compared with rates during first COVID-19 wave (CW 10–20/2020), first loosening of restrictions (CW 21–29/2020), intensified COVID-19 testing (CW 30–39/2020) and second COVID-19 wave (CW 40–53/2020), and contrasted with COVID-19 incidence in Germany.

**Results:**

A total of *n* = 10 810 NECs were analyzed. Prepandemic NEC rates were stable over time (median: 103, IQR: 97–115). Upon the first COVID-19 wave in Germany, NEC rates declined sharply (median: 86, IQR: 69–92; *p* < 0.001) but recovered within weeks. Changes in NEC rates after first loosening of restrictions (median: 109, IQR: 98–127; *p* = 0. 188), a phase of intensified testing (median: 111, IQR: 101–114; *p* = 0.434) and as of a second COVID-19 wave (median: 102, IQR: 94–112; *p* = 0. 462) were not significant. Likewise, patient age and gender distribution remained constant.

**Conclusion:**

Upon the first pandemic COVID-19 wave in Germany, NEC rates declined but recovered within weeks. It is unknown whether this recovery reflects improved medical care and test capabilities or an adjustment of the patients’ behaviour.

**Supplementary Information:**

The online version contains supplementary material available at 10.1186/s42466-021-00147-8.

## Background

In calendar week (CW) 11 in 2020, the World Health Organization declared the outbreak of the severe acute respiratory syndrome coronavirus 2 (SARS-CoV-2) a pandemic [1]. Since then, states worldwide have resorted to measures aimed at social distancing to prevent or protract the spread of the coronavirus, including stay-at-home orders.

To enable comprehensive patient care in emergency situations, the AHA/ASA Stroke Council Leadership, for example, issued a temporary emergency guidance to US stroke centers during the coronavirus disease 2019 (COVID-19) pandemic on 1 April 2020 [2]. The recommendations and anecdotal advice offered provide information how to deliver stroke care and how to protect others and oneself from being infected. A key point in providing care, however, could not be fully anticipated and covered – the dynamics of the pandemic and its influence on emergency consultations.

There are reports from various specialties (e.g. psychiatry, dermatology) from different countries in which a decrease in emergency consultations was noted within the first weeks of the COVID-19 pandemic [3, 4]. This also includes a significant decrease in patients treated with mechanical thrombectomy for acute ischemic stroke [5]. However, these reports only relate to the first few weeks of the pandemic, in which this drastic event imposed changes and challenges to adapt access to all levels of medical care.

The purpose of this study is to analyze our referrals during the first year of the COVID-19 pandemic in a tele-radiological network and discuss the potential impact on care for neurological emergencies requiring imaging.

## Methods

We performed an ambispective data collection of a tertiary care university hospital with a comprehensive stroke center and permanent neuroradiological and neurointerventional attendance. Our department provides neuroradiological emergency consultations in a teleradiological network covering 13 hospitals in nine districts in the Federal States of Baden-Württemberg, Hesse and Rhineland-Palatinate in southwest Germany (total population 1 916 000; Table 1). Our department provides neuroradiological emergency consultations (NECs) for computed tomography imaging for these hospitals from 5 PM to 8 AM on weekdays and 24 h on weekends and holidays.

**Table 1:** State, district and number of hospitals covered by the teleneuroradiological network

State	District	Number of hospitals covered
Baden-Württemberg	Hohenlohekreis	1
Baden-Württemberg	Neckar-Odenwald-Kreis	2
Baden-Württemberg	Rhein-Neckar-Kreis	4
Hesse	Kreis Bergstraße	1
Hesse	Landkreis Darmstadt-Dieburg	1
Hesse	Main-Taunus-Kreis	1
Hesse	Odenwaldkreis	1
Hesse	Rheingau-Taunus-Kreis	1
Rhineland-Palatinate	Landkreis Bad Kreuznach	1

### Patients, imaging acquisition and interpretation

The teleradiological network cares for patients presenting with different neurological emergencies, reaching from trauma, brain tumors, unclear loss of consciousness, and most importantly stroke. Patients who appear in an emergency department of the remote hospitals within the teleradiological network are examined clinically by the medical staff on site. In patients who show neurological symptoms or in suspected affection of the brain or spine, non-contrast-enhanced computed tomography (NCCT) of the brain, spine and, if indicated, contrast-enhanced imaging of the vessels supplying the brain (CT-angiography, CTA) is arranged after consultation with the neuroradiologist. Image acquisition and reconstructions are being performed by a radiology-technician within the remote hospitals. Images are transferred via a secure line and interpreted by a neuroradiologist at our department. The radiological findings are discussed by telephone with the referring physician and subsequently a written report is sent.

We report descriptive data with reference to the number of performed NECs within our teleradiological network in the first year of the COVID-19 pandemic.

In accordance with the recommendation of the Robert Koch-Institute concerning the definition of phases to describe the COVID-19 pandemic in Germany 2020 [6], the study period was per calendar weeks (CW) divided into 5 phases:

- pre-pandemic phase with sporadic COVID-19 cases (PP, CW: 01/2019–9/2020, i.e. 30 December 2019 - 1 March 2020),
- phase 1: first COVID-19 wave and 1st pandemic lockdown (CW: 10–20/2020, i.e. 2 March 2020 - 17 May 2020),
- phase 2: a (gradual loosening of anti-coronavirus measures; CW: 21–29/2020, i.e. 18 May 2020 - 19 July 2020) and b (intensified COVID-19 testing; CW: 30–39/2020, i.e. 20 July 2020 - 27 September 2020),
- phase 3: second COVID-19 wave and 2nd pandemic lockdown (CW: 40–53/2020, i.e. 28 September 2020 - 3 January 2021).

Weekly incidence rates of COVID-19 in Germany were obtained from the Robert Koch-Institute (RKI: SurvStat@RKI 2.0, https://survstat.rki.de, query date: 13 February 2021).

### Statistical analysis

Statistical analysis was performed by using SPSS Statistics (21.0.0.0; IBM, Armonk, NY). Continuous variables are presented as means and SD or medians and interquartile intervals, and categorical variables as absolute values and percentages. A two-sided *P*-value of 0.05 was considered statistically significant.

## Results

In this study, a total of *n* = 10 810 NECs were analyzed. Gender was male in *n* = 5299 patients (49%), female in *n* = 5460 patients (50.5%), and unknown in *n* = 51 patients (0.5%). Mean patient age was 73 (SD: 16) years. Patient age, as well as age and gender distribution remained stable throughout the different pandemic phases (Supplemental Figure S1, Supplemental Figure S2 and Supplemental Figure S3).

In the pre-pandemic period, a median of 103 consultations per week was called (interquartile range: 97–115). After Germany encountered the phase 1, i.e. first COVID-19 wave and entered the 1st pandemic lockdown, NECs declined sharply (median: 86, IQR: 69–92; *p* < 0.001), followed by a rebound until the end of the first COVID-19 lockdown in CW 20. This represents a decrease by 16.5% compared to the pre-pandemic period.

**Fig. 1:**
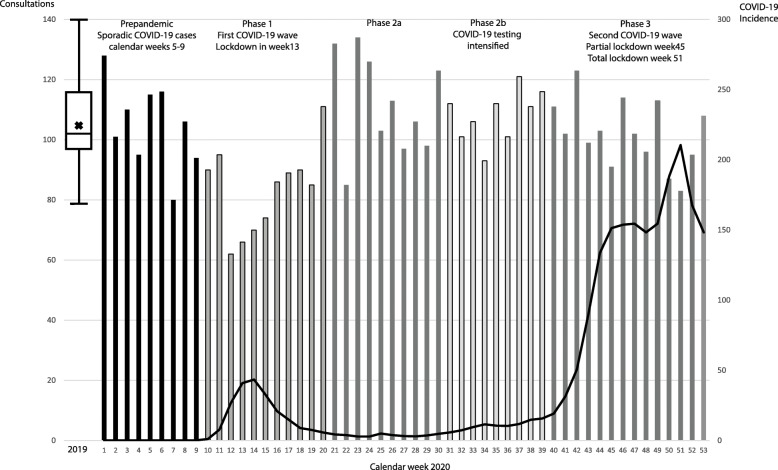
Number of neuroradiological emergency consultations in a teleradiological network (bars) and 7-day incidence of notified SARS-CoV-2 infections* (line) by calendar week and pandemic lockdown phase in Germany, 2019 (boxplot) and calendar weeks 01–53/2020. *Source: Robert Koch-Institute

Following gradual loosening of anti-coronavirus measures during the summer of 2020, the number of NECs resumed to a level numerically slightly above the previous year (phase 2 a: median: 109, IQR: 98–127, *p* = 0.188; phase 2b: median 111, IQR: 101–114; *p* = 0.434). With increasing COVID-19 case numbers Germany encountered a second COVID-19 wave and contact limits and anti-coronavirus measures were tightened again. During the second wave (phase 3), NECs per week adjusted to a level that was almost identical to that of the pre-pandemic period (median: 102, IQR: 94–112; *p* = 0.462; Fig. 1 and Supplemental Figure S4).

**Fig. 2:**
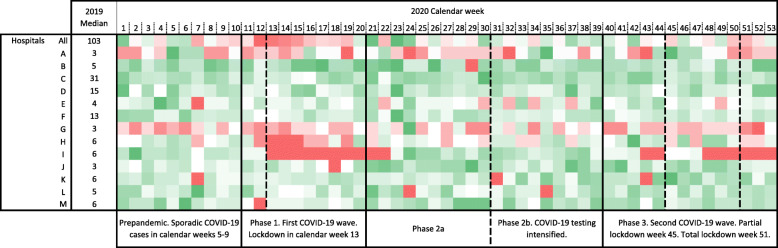
Heatmap showing the number of neuroradiological emergency consultations (NECs) per calendar week in a teleradiological network for 13 hospitals compared to the reference value (median) in the year 2019. Red: number of NECs below reference value. Green: number of NECs exceeds reference value. White: number of NECs is the same as the reference value

A direct comparison of the number of NECs performed per calendar week in 2020 to the annual median from 2019 shows a decline when the first pandemic lockdown was entered. It is noticeable that although there are individual differences, this affects most of the hospitals connected to the teleradiology network in the first lockdown (Fig. 2). A divergent picture can be observed after phase 1, i.e. the first lockdown. While there were individual hospitals that had requested their original or a higher number of NECs (e.g. hospital C, K and M), a reduced number of NECs was performed for other hospitals (e.g. hospital A, G, H and I). With the tightening of the anti-coronavirus measures and again with the entry into the second pandemic lockdown, the number of NECs per hospital decreased once again, but less sharply and to different degrees.

## Discussion

We report on the variation of neuroradiological emergency consultations in a teleradiological network that covers 13 hospitals in the southwest of Germany during the first year of the SARS-CoV-2 pandemic. After a strong decline during the first pandemic lockdown, the number of NECs numerically exceeded the previous year’s level upon gradual loosening of anti-coronavirus measures. The later observation lasted until COVID-19 cases rose dramatically in Germany, which led to tightening of contact restrictions and to a second pandemic lockdown by the end of 2020. After a second nationwide hard lockdown was imposed, NECs did not fall below the previous year’s level or to the level during the first lockdown.

The 16.5% decline of NECs during the first pandemic lockdown cannot be explained by seasonal changes or equipment downtimes. The decline in the number of NECs concurs with the onset of nationwide containment measures in CW 12 of 2020, which included stay-at-home recommendations and physical distancing. Our observation coincides with reports from different countries and various specialties including emergency department utilization (decline by 29%), stroke (decline by 20%) and mechanical thrombectomy (decline by 21%) [3–5, 7–11]. However, these reports portrayed only the initial impact phase of the COVID-19 pandemic. This phase was characterized worldwide by an insufficient supply of personal protective equipment, the establishment of coronavirus test capacities for the first time, a sometimes extraordinarily increased workload, possibly locally even overload of the health system and a lack of fundamental knowledge about the disease. In this phase, in addition to following stay-at-home orders, the fear of infection with the coronavirus within a hospital may have led to a reduction in seeking medical care and subsequently in NECs in our teleradiological network [12, 13].

Soon after the first few weeks of the pandemic and a decrease of incident infections, anti-coronavirus measures were loosened in Germany. As it was evident that the pandemic would last for several months, potentially years, adaptation to the situation - the so-called *new normal* – occurred. Patient care was reorganized in compliance with distance regulations and increased acceptance and use of telemedicine technologies [14, 15]. The numerical increase, i.e. the return to the previous year’s level in the NEC in the phase after the first lockdown might be caused by i) the “normal” need of patients to seek medical attention, ii) a response from the population to requests from healthcare professionals to seek medical attention in the event of (mild) clinical symptoms (e.g. suggestive of transient ischemic attacks), and iii) improved acceptance and use of telemedicine by healthcare professionals [15, 16].

The renewed, less pronounced decrease of the NECs in our teleradiological network during lockdown light and the second pandemic lockdown in Germany appear to indicate that the initial fear present during the first lockdown seems to be overcome and the motivation to choose not to come to the hospital has reduced. This might be the result of the increasing knowledge about the disease, an improved supply with personal protection gear and the circumstance that patients and hospitals have adapted better to the pandemic situation – i.e. to the *new normal*.

Our observations stand to some extent in contrast to observations made from hospital admissions through emergency departments in the United States in calendar weeks 11 through 36 (11 March 2020 8 September 2020) [8]. However, as incidence of COVID-19 cases and applicable contact restrictions were inhomogeneous and differ to our catchment area, the prolonged decline in emergency admissions described by Nourazari et al. might not be transferable to our setting. At this point, a comparison of the countries seems only possible to a limited extent due to different health systems, anti-coronacirus measures and incidences. Based on our data, it could be assumed that with appropriate measures, the number of emergency treatments in other settings would also increase to the previous year’s level.

Based on the assumption that the incidence of stroke and other neurological emergencies is stable and that there were no major staff fluctuations in the affiliated hospitals, there are different possibilities that could explain the observed decline in neuroradiological emergency consultations. The reduction in consultations might also be explained by a temporary excessive workload of hospital personnel. Healthcare professionals are struggling to provide care for patients with COVID-19 and try to maintain their health in the doing [17, 18]. The “Temporary emergency guidance to US stroke centers” addresses this issue to some extent.

Patients might choose not to seek emergency medical care and forgo a proper workup of neurological symptoms [19]. Fear of being infected with SARS-CoV-2 during a visit to the hospital or the doctor may have contributed to the massive reduction of NECs during the first pandemic lockdown. This presumed effect was probably less pronounced during the lockdown light or the second pandemic lockdown. It seems understandable that patients with transient or mild symptoms avoid going to the hospital to avoid the risk of contracting COVID-19 and to protect others. In the face of an impending recession triggered by the pandemic, possible or already occurring economic consequences will be another aspect that leads to patients with transient or mild symptoms not visiting a doctor [20].

However, minor or transient symptoms are a major warning and may signal a severe stroke to come in the future. As contact restrictions and other anti-coronavirus measures might last for months or occur repeatedly due to mutations of the virus and local spread of infections, several patients might not get the appropriate diagnostic workup and treatment for TIA and other neurological diseases in time. Particularly affected will probably be persons who are at a higher risk to suffer from stroke and at higher risk for severe illness from COVID-19, i.e. people of higher age, people who have serious heart conditions and people with diabetes [21, 22]. Richter et al. reported a significant decrease in hospitalization of acute ischemic stroke, transient ischemic attack, and intracerebral hemorrhage patients, whereas mechanical thrombectomy rate was significantly higher during the first pandemic (16 March to 15 May 2020) compared with the prepandemic period in Germany [23].

The reasons for the decline of neuroradiological emergency consultations require further research. We are concerned that the current high load on the medical system caused by the COVID-19 pandemic may disguise the underdiagnosis and delay in treatment of neurologic emergencies, i.e. stroke. The pandemic’s collateral damage may therefore include a higher individual and societal burden of lifelong disability or dependence, resulting from the underdiagnosis and delay in the treatment of stroke.

Our study has some limitations. First, access to the individual patient files was limited. This study only used data on neuroradiological emergency consultations performed during the observation period. Therefore, no differentiated picture of the diagnoses underlying hospital admission can be obtained. Second, as we do not have full access to admission records it cannot be answered why fewer neuroradiological emergency consultations were requested because 1.) fewer patients came to the hospitals, ii) the staff did not request neuroradiological emergency consultations, i.e. CT imaging, in order to make the patient’s stay in the emergency department as short as possible, or iii) health services were reorganized so that patients with neurological symptoms primarily presented to other hospitals outside our catchment area. Due to timely limited reorganization of local emergency medical care, patients with neurological emergencies were not admitted to hospital I (Fig. 2), but primarily transferred from the emergency medical services to neighboring hospitals outside of our catchment area. Third, seasonality linked to public holidays in November and December must also be considered as possible influencing factors on the demand for medical care. Since clinical signs of neurological emergencies are usually deeply disturbing to the patient and persons in direct contact and are sometimes accompanied by a change in the level of consciousness, seasonal influence should be minimal [24, 25]. Last, as hospital practices and guidelines have varied across different countries, our results might not necessarily reflect observations made in other settings. However, as a decrease in diagnosis, staging, and treatment for other diseases was observed in the same period of the COVID-19 pandemic [10], our results are probably transferrable to a certain extent.

## Conclusion

Upon the first COVID-19 pandemic lockdown, neuroradiological emergency consultations declined but recovered within weeks. This observation has clinical implications as the time window and opportunities for appropriate diagnostic work-up and treatment for some patients may have been missed. No decrease in the NECs was observed during the summer or the second COVID-19 wave. It is unknown whether the recovery reflects improved medical care and test capabilities or an adjustment of the patients’ behaviour.

## Supplementary Information

**Additional file 1: Supplemental Figure S1.** Age of patients who received a neuroradiological emergency consultation in a teleradiological network by pandemic phase, Germany 2019–2020. For timeline of pandemic phases see full paper. **Supplemental Figure S2.** Age distribution of patients who received a neuroradiological emergency consultation in a teleradiological network by pandemic phase, Germany 2019–2020. **Supplemental Figure S3.** Gender distribution of patients who received a neuroradiological emergency consultation in a teleradiological network by pandemic phase, Germany 2019–2020. **Supplemental Figure S4.** Number of neuroradiological emergency consultations per calendar week in a teleradiological network (boxplots) by pandemic phase, Germany, 2019–2020.

## Data Availability

The data that support the findings of this study are available from the corresponding author upon reasonable request.

## Abbreviations

COVID-19: Coronavirus disease 2019
CW: Calendar week
NCCT: Non-contrast-enhanced computed tomography of the head
NEC: Neuroradiological emergency consultation
SARS-CoV-2: Severe acute respiratory syndrome coronavirus 2
