# Supplementary material for: Neuroradiological emergency consultations during the first year of the COVID-19 pandemic
Source: Neurol Res Pract. 2021 Aug 30;3:47. doi: 10.1186/s42466-021-00147-8 (PMC8403519; doi:10.1186/s42466-021-00147-8)
Supplement: Supplementary file 1 — Additional file 1: Supplemental Figure S1. Age of patients who received a neuroradiological emergency consultation in a teleradiological network by pandemic phase, Germany 2019–2020. For timeline of pandemic phases see full paper. Supplemental Figure S2. Age distribution of patients who received a neuroradiological emergency consultation in a teleradiological network by pandemic phase, Germany 2019–2020. Supplemental Figure S3. Gender distribution of patients who received a neuroradiological emergency consultation in a teleradiological network by pandemic phase, Germany 2019–2020. Supplemental Figure S4. Number of neuroradiological emergency consultations per calendar week in a teleradiological network (boxplots) by pandemic phase, Germany, 2019–2020. [file 42466_2021_147_MOESM1_ESM.docx]

Neuroradiological emergency consultations during the first year of the COVID-19 pandemic

## Data Supplement (Online)

# Supplemental Figures:


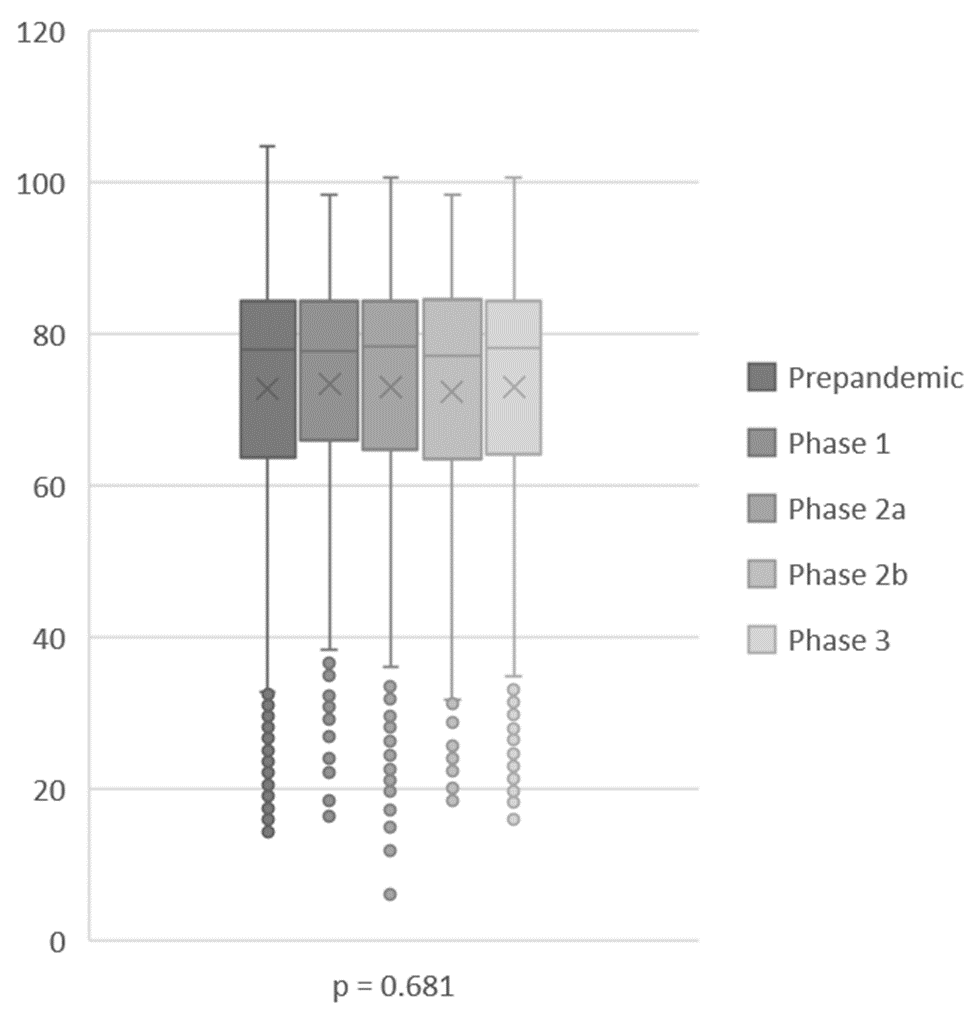


Supplemental Figure S1. Age of patients who received a neuroradiological emergency consultation in a teleradiological network by pandemic phase, Germany 2019-2020. For timeline of pandemic phases see full paper.


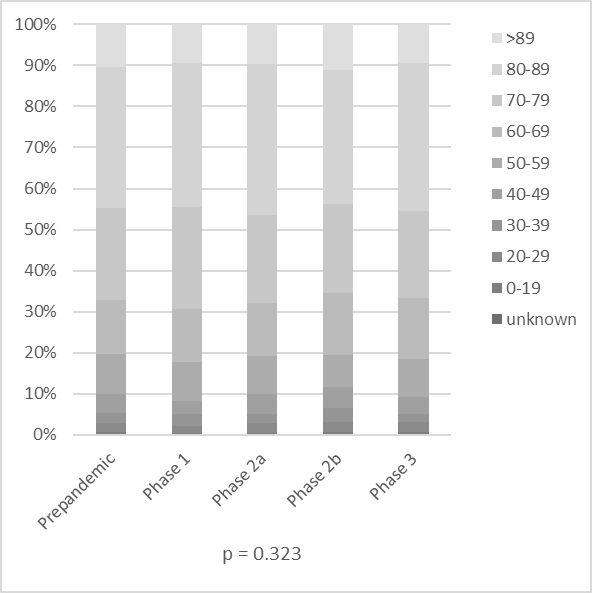


Supplemental Figure S2. Age distribution of patients who received a neuroradiological emergency consultation in a teleradiological network by pandemic phase, Germany 2019-2020.


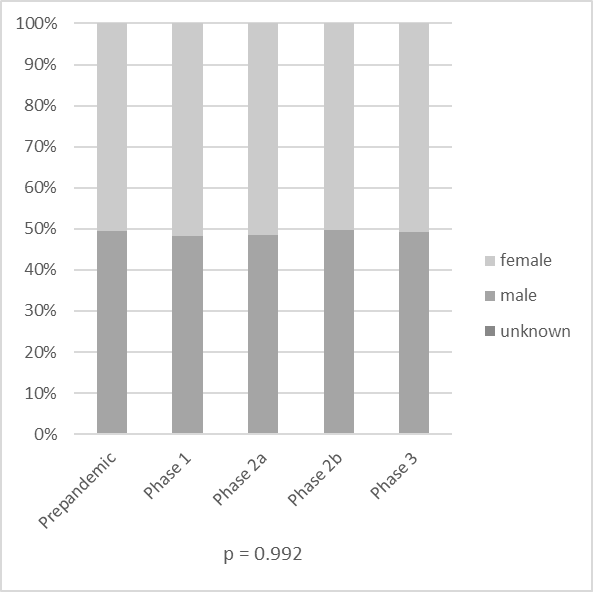


Supplemental Figure S3. Gender distribution of patients who received a neuroradiological emergency consultation in a teleradiological network by pandemic phase, Germany 2019-2020.


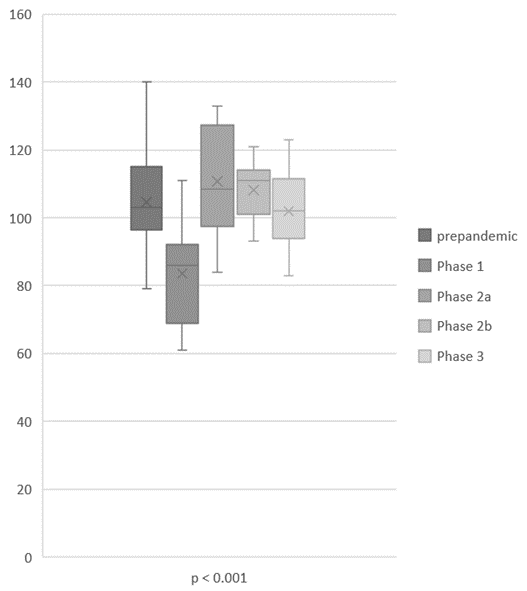


Supplemental Figure S4. Number of neuroradiological emergency consultations per calendar week in a teleradiological network (boxplots) by pandemic phase, Germany, 2019-2020
